# Sex partnerships, health, and social risks of young men leaving jail: analyzing data from a randomized controlled trial

**DOI:** 10.1186/1471-2458-10-689

**Published:** 2010-11-10

**Authors:** Megha Ramaswamy, Nicholas Freudenberg

**Affiliations:** 1 Department of Preventive Medicine and Public Health, University of Kansas School of Medicine, Kansas City, Kansas, USA; 2 Department of Public Health, City University of New York School of Public Health at Hunter College, New York, New York, USA

## Abstract

**Background:**

Young men involved in the criminal justice system face disproportionately high rates of sexual risk behavior, drug, use, and violence. Little is known about how their involvement in sex partnerships might mitigate their unique health and social risks. This study explores whether sex partner experience protects against harmful sexual behaviors, drug problems, violence, and recidivism in 16-18-year-old Black and Latino men leaving a US jail.

**Methods:**

Data were drawn from the Returning Educated African-American and Latino Men to Enriched Neighborhoods (REAL MEN) study conducted between 2003-2007, which tracked 552 adolescents during their time in a New York City jail and 397 of them one year after their release. Logistic regression was used to examine the relationship between sex partner experience and sex behavior, drug use, violence, and recidivism.

**Results:**

This study indicates that young men who have long-term sex partners prior to incarceration are less likely to be inconsistent condom users (OR = 0.50, p ≤ 0.01), have sex while high on drugs/alcohol (OR = 0.14, p ≤ 0.001), use marijuana daily (OR = 0.45, p ≤ 0.001), and carry weapons during illegal activity (OR = 0.58, p ≤ 0.05), especially compared with peers who simultaneously are involved with long-term and casual "short-term" sex partners. However, the positive effects of having a long-term sex partner generally do not apply over time - in this case, one year after being released from jail. Aside from sexual partners, factors such as employment and housing stability predict whether these young men will experience positive or negative outcomes post-incarceration.

**Conclusions:**

This study highlights the importance and potential benefits of health interventions that engage young Black and Latino men who are involved in the criminal justice system in the US, as well as their sex partners, in health promotion programs. The study also confirms the need for programs that address the employment and housing needs of young men after they leave correctional facilities.

## Background

In this study, we explore the role of sex partnerships in the health and social risks of young men leaving jail. Research consistently demonstrates that compared with non-incarcerated youth in the US, incarcerated adolescents face disproportionately high health and social risks related to sex, drugs, violence, and recidivism [1-4]. Most interventions designed to mitigate these risks operate at the behavioral level [5] and rarely lead to significant or lasting behavioral changes - and therefore improved health and social outcomes - in the long term. Few of the prior studies seriously consider the romantic and sexual relationships of adolescents, a matter central to their lives.

Interestingly, recent studies of adolescent relationships among non-adjudicated youth confirm that both romantic and non-romantic sexual relationships influence a teen's sex choices, drug use, crime, and educational involvement - research that appears to confirm what we propose as a correlation between sexual partnership arrangements and risk behavior [6-8]. However, these latter studies fail to place these findings in the social context in which urban Black and Latino young men in the US live. For example, these youth generally face significant race, gender, educational, and economic barriers to success [9-12], which add to the burden of a criminal history that youth in the present study carry. Studies of disadvantage, health, and social outcomes among poor Black and Latino young men [13-15] also usually fail to consider the role that sex partners play in these young men's lives.

This study investigates whether sex partner experience can mitigate high-risk health and social behaviors by taking into account this prior research on risk factors and interventions for incarcerated adolescents, studies on teen relationships, and an understanding of the social context in which urban Black and Latino youth involved in the criminal justice system live. Should sex partner experience serve as an important protective or risk-enhancing factor, then interventions designed to engage young people will certainly need to include discussions about sex partnerships.

## Methods

This study is based on data collected as part of the REAL MEN study (Returning Educated African-American and Latino Men to Enriched Neighborhoods - data collection supported by the National Institute of Drug Abuse R01 DA014725 Impact of an HIV Intervention on Adolescent Males Leaving Jail), which was a randomized controlled trial designed to assess the impact of an intervention for young men leaving New York City jails. Its goals were to reduce drug use, risky sexual behavior and criminal activity among 16 to 18-year-old males. Participants were recruited from two facilities located at the New York City Department of Correction's Rikers Island Detention Center that house all New York City male adolescent inmates.

After an informed consent process, the research team enrolled 552 young men in the study. In general, the sample was representative of the overall adolescent population leaving New York City jails in its racial/ethnic and criminal history characteristics. The intake interviews were conducted in the jail by project staff and included questions on demographic, educational and employment histories, as well as criminal justice involvement, substance use, HIV awareness, sexual behavior, and attitudes about racial/ethnic identity and gender norms. After the interview, participants were randomly assigned to receive either a jail-based discharge planning session or a 30-hour intervention that began in jail and continued in the community after release. Although the goal of the present study is not to evaluate this intervention, results presented here will take into account the effect of the intervention. (A description of the intervention and results from the evaluation can be found elsewhere [5,16].) Follow-up Time 2 interviews with enrolled participants were conducted at approximately 12 months after release from jail. This interview was completed by 397 participants - a follow-up rate of 72%. The majority of participants completed the follow-up interview in a research office, while the rest were interviewed in a New York City jail, by telephone, in a state prison or in some other setting. All participants completed an informed consent document approved by Hunter College and the New York City Department of Health and Mental Hygiene Institutional Review Boards.

### Variables

The key independent variable of interest was a categorical variable designed to assess sex partner experience in the three months prior to the initial incarceration and one year after release from jail. The following three categories were analyzed: (1) having had a long-term sex partner only, defined as a partner for at least three months and for whom participants had romantic feelings; (2) having had a short-term sex partner only, defined as someone participants had sex with that they just met, someone they'd been with for less than three months, someone participants didn't know very well but had sex with occasionally, or causal sex partners; and finally (3) having had both long-term and short-term sex partners simultaneously. For multivariate analysis, each category was recoded into a dummy variable to include the following comparisons: (1) having had a short-term sex partner only, compared to having had a long-term sex partner only; (2) having had both long-term and short-term sex partners simultaneously compared to having had only a long-term sex partner. For this study, we were primarily interested in the benefits of having a long-term sex partner compared to other types of partnerships.

The primary dependent variables were both continuous and dichotomous variables that assessed behavioral and criminal justice characteristics both prior to incarceration and one year after release from jail. The following variables were used to measure sex risk: having had three or more sex partners in the past three months, using condoms inconsistently with all sex partners in the past three months, and being under the influence of drugs or alcohol more than half the time during intercourse with all sex partners in the past three months. To measure drug risk, we examined the following variables: drug and alcohol dependence in the past year based on DSM IV criteria, daily marijuana use in the past 30 days (prior to incarceration) or past 90 days (one year after release), and any hard drug use in the past 30 days (prior to incarceration) or past 90 days (one year after release). The following variables were examined to measure violence: carrying weapons during illegal activity in the past year, sustaining an injury due to violence in the past year, or having a current violent charge on record (only measured prior to incarceration). Finally, we used the following variables to measure recidivism one year after release from jail: any further arrests or a subsequent incarceration.

All multivariate analysis included controls for the following variables: age, Black or Hispanic race/ethnicity, number of previous arrests, days incarcerated between index and follow-up interview, randomization to the intervention that was part of the parent study (to assess whether the intervention had any effect on outcomes of interest in this paper), not attending school, ever being held back, not being employed, not living with parents, and having an unstable living situation. For multivariate analysis, each category for all dichotomous dependent and control variables was recoded into a dummy variable.

### Data Analysis

We generated descriptive data on key variables of interest. Variables were chosen for multivariate analysis based on bivariate analyses (not shown here) and the literature on social context for health risk. We used logistic regression [17] to measure the relationship between sex partner experience and the four key outcomes: high-risk sexual activity, drug use problems, violent behavior, and recidivism. Three primary regression analyses were conducted: (1) to assess the relationship between sex partner experience prior to incarceration and the key outcomes (outlined above) prior to incarceration; (2) to assess the relationship between sex partner experience prior to incarceration and key outcomes one year after release from jail; and (3) to assess the relationship between sex partner experience one year after release from jail and key outcomes one year after release from jail. All analyses were conducted with SPSS Version 18 (SPSS Inc., Chicago, Ill).

## Results

Table 1 provides an overview of participant characteristics and key variables both prior to incarceration and one year after release from jail. All participants were male, and the mean age at the time of the intake interview was 17.99 (SD 0.71). One year after release from jail, the mean age of participants was 19.60 (SD 0.93), suggesting that on average young men spent seven months in jail prior to release. The majority of the follow-up sample was Black (55.8%) or Latino (38.1%).

**Table 1:** Variables of interest prior to incarceration and one year after release from jail

	Prior to Incarceration (N = 552)	One Year After Release from Jail (N = 397)
Age	Mean = 17.99*	Mean = 19.60
Black	57.4%	55.8%
Hispanic	37.4%	38.1%
Previous arrests	Mean = 5.23	--
Days incarcerated in last year	--	Mean = 84.92
Randomized to intervention	--	49.4%
Not in school	67.4%	79.7%
Ever held back	46.6%	--
Not employed	77.9%	77.0%
Not living with parents	12%*	37.9%
Unstable living situation	2.5%*	24.8%
3+ sex partners	43.1%*	4.1%
Inconsistent condom use	68.5%	83.6%
Sex while high	60.3%	69.1%
Drug/alcohol dependence	22.3%*	19.6%
Daily marijuana use	54.7%*	31.0%
Any hard drug use	6.5%*	11.3%
Weapons possession	63.9%*	28.6%
Any violent injury^a^	54.6%	50.9%
Incarcerated for violent charges	37.1%	--
Rearrested	--	67.5%
Went back to jail	--	46.9%
Long-term sex partners only	24.1%*	37.0%
Short-term sex partners only	21.3%	21.8%
Both long/short-term partners at same time	54.5%*	41.3%

*p ≤ 0.05

About one-quarter of participants (N = 130, 24.1%) reported having a long-term sex partner prior to incarceration. One hundred and fifteen participants (21.3%) reported having a short-term sex partner only, and 294 participants (54.5%) said they had both types of partners simultaneously. One year after the young men were released from jail, 112 of the men (37.0%) said they had long-term sex partners only, 66 (21.8%) reported short-term partners only, and 125 (41.3%) reported both types of partners simultaneously. In analysis of the Chi-Square statistic, sex partner experience prior to incarceration and one year after release is, in fact, significantly different (p ≤ 0.05). This indicates that patterns of sex partners (and sex partners) do change over time, particularly after an incarceration experience for one of the partners, in part due to the disruptive effect of incarceration and in part to normal adolescent development.

### Multivariate Analysis of Sex Partner Experience

In logistic regression analysis, the type (i.e., short or longer term) of sex partners these young men experienced prior to incarceration was related to the number of sex partners, condom use, having sex while high, marijuana use, and carrying weapons during illegal activity prior to incarceration. Young men who reported having both types of sex partners simultaneously had increased risk of engaging in these risk behaviors. For each of these indicators, the regression models had good fit (see Table 2 for model fit, odds ratios, and logistic regression coefficients). Additionally, those young men who reported having short-term sex partners only were also more likely to have had three or more sex partners (OR = 28.37, p ≤ 0.001), have had sex while high on drugs/alcohol (OR = 3.51, p ≤ 0.001), and used marijuana daily (OR = 2.09, p ≤ 0.01), compared to those men with long-term sex partners only prior to incarceration (results not shown in tables).

**Table 2:** Sex, drug, and violence outcomes by sex partner experience prior to incarceration, N = 552

	OUTCOMES PRIOR TO INCARCERATION, OR (B)								
	**3+ sex partners**	**Inconsistent condom use**	**Sex while high on drugs/** **alcohol**	**Drug/alcohol dependence**	**Daily marijuana use**	**Any hard drug use**	**Weapons possession during illegal acts**	**Any violent injury**	**Incarcerated for violent charge**

INDEPENDENT VARIABLES AND CONTROLS PRIOR TO INCARCERATION									
Both long/short-term partners simultaneously^†^	27.80 (3.33)***	2.01 (0.70) **	7.00 (1.95) ***	1.27 (0.24)	2.20 (0.79)***	0.74 (-0.30)	1.72 (0.54)**	1.27 (0.24)	1.45 (0.37)
Age	0.85 (-0.17)	1.02(0.02)	0.95 (-0.06)	0.86 (-0.15)	0.96 (-0.04)	1.50 (0.41)	0.83 (-0.19)	1.17 (0.16)	0.79 (-0.23)
Black	2.69 (0.99)	0.60 (-0.51)	1.60 (0.47)	0.62 (-0.47)	2.36 (0.86)	0.16 (-1.85)**	1.45 (0.37)	0.96 (-0.05)	0.65 (-0.43)
Hispanic	3.07 (1.12)*	0.76 (-0.27)	0.95 (-0.05)	0.98 (-0.02)	1.90 (0.64)	0.23 (-1.48)*	1.76 (0.56)	1.09 (0.09)	0.57 (-0.56)
Previous arrests	1.04 (0.04) ***	1.02 (0.02)	1.09 (0.08) **	1.00 (-0.00)	1.05 (0.05)*	1.01 (0.01)	1.09 (0.09)***	1.02 (0.02)	0.97 (-0.03)
Not in school	1.52 (0.42) ***	1.16 (0.15)	1.41 (0.34)	2.78 (1.02) ***	1.29 (0.26)	1.21 (0.19)	0.84 (-0.18)	1.35 (0.30)	0.81 (-0.21)
Ever held back	1.08 (0.08)	0.87 (-0.14)	1.25 (0.22)	1.03 (0.03)	1.20 (0.19)	2.46 (0.90)*	1.11 (0.10)	0.77(-0.27)	0.91 (-0.09)
Not employed	0.92 (-0.08)	1.09 (0.09)	1.54 (0.43)	1.19 (0.18)	1.23 (0.20)	1.12 (0.11)	1.23 (0.24)	1.31 (0.27)	0.81 (-0.21)
Not living with parents	1.04 (0.04)	2.54 (0.93)*	2.13 (0.76)	0.89 (-0.12)	1.42 (0.35)	1.38(0.32)	0.94 (-0.06)	1.56 (0.46)	0.70 (-0.35)
Unstable living situation	1.03 (0.03)	0.45 (-0.79)	0.60 (-0.51)	4.54 (1.51)	2.12 (0.72)	14.15 (1.42)	1.73 (0.55)	0.47 (-0.76)	1.68 (0.52)
Omnibus test of model coefficients χ2 (p value)	124.74 (0.00)	17.30 (0.06)	91.20 (0.00)	23.65 (0.01)	29.20 (0.00)	16.48 (0.09)	24.80 (0.01)	3.28 (0.97)	12.97 (0.23)
Nagelkerke R2	0.36	0.06	0.28	0.09	0.09	0.10	0.07	0.03	0.04

*p ≤ 0.05, **p ≤ 0.01, ^*** ^p ≤ 0.001

^† ^vs. Both long/short-term partners at same time vs. long-term partner only

In an analysis of sex partner experience prior to incarceration and outcomes *one year after release from jail *(not shown in tables), we found that young men who had both types of sex partners simultaneously were twice as likely to be daily marijuana users (OR = 2.31, p ≤ 0.05) compared to those men with long-term sex partners only. There were no other statistically significant relationships between either type of sex partner experience and the remaining outcomes.

When analyzing sex, drug, violence, and recidivism outcomes one year after release from jail by sex partner experience in that interval (see Table 3), having both types of sex partners simultaneously was inversely related to engaging in intercourse while high on drugs/alcohol. Other factors, namely post-incarceration unemployment and housing instability, predicted health and social outcomes one year after release from jail.

**Table 3:** Sex, drug, and violence outcomes by sex partner experience one year after release from jail, N = 397

	OUTCOMES ONE YEAR AFTER RELEASE FROM JAIL, OR (B)									
	**3+ sex partners**	**Inconsistent condom use**	**Sex while high on drugs/alcohol**	**Drug/alcohol dependence**	**Daily marijuana use**	**Any hard drug use**	**Weapons possession during illegal acts**	**Any violent injury**	**Rearrest**	**Went back to jail or a prison**

INDEPENDENT VARIABLES AND CONTROLS (EXCEPT AGE, RACE) ONE YEAR AFTER RELEASE FROM JAIL										
Both long/short-term partners simultaneously^†^	31.23 (1.14)	0.00 (-20.12)	0.13 (-2.08)**	1.06 (0.06)	1.65 (0.49)	0.98 (-0.03)	1.92 (0.65) ^‡^	0.28 (-1.27)	1.16 (0.12)	1.00 (-0.00)
Age	1.54 (0.43)	1.40 (0.33)	1.74 (0.56)*	1.64 (0.49)	1.79 (0.51)*	1.90 (0.64)	1.31 (0.27)	1.38 (0.32)	1.54 (0.34)	0.90 (-0.15)
Black	0.06 (-2.89)*	0.44 (-0.82)	2.12 (0.77)	0.86 (-0.15)	1.76 (0.28)	0.62 (-0.47)	0.43 (-0.85)	2.08 (0.73)	0.85 (-0.14)	0.45 (-0.61)
Hispanic	0.06 (-2.78)*	0.50 (-0.69)	2.91 (1.07)	0.68 (-0.38)	1.75 (0.54)	0.78 (-0.25)	0.70 (-0.36)	1.58 (0.45)	1.08 (0.01)	0.69 (-0.50)
Days incarcerated in last year	0.99 (-0.01)	1.00 (0.00)	1.00 (-0.00)	1.00 (0.00)	1.00 (-0.00)	1.00 (-0.00)	1.00 (0.00)	1.00 (0.00)	1.00 (0.00)	1.00 (0.01)***
Randomized to intervention	1.67 (0.51)	1.00 (0.00)	0.79 (-0.23)	0.79 (-0.24)	1.00 (-0.06)	1.12 (0.17)	1.15 (0.14)	1.33 (0.28)	0.77 (-0.24)	1.13 (0.13)
Not in school	0.49 (-0.71)	0.56 (-0.58)	1.51 (0.41)	1.34 (0.29)	1.89 (0.76)	0.67 (-0.41)	1.16 (0.15)	0.40 (-0.93)	1.45 (0.38)	2.00 (0.59)
Not employed	0.00 (18.65)	0.88 (-0.13)	1.94 (0.66)	2.12 (0.77)	5.31 (0.97)***	1.52 (0.42)	1.15 (0.14)	3.10 (1.13)	1.35 (0.39)	1.23 (0.21)
Not living with parents	1.86 (0.59)	0.00 (19.54)	0.71 (-0.35)	0.43 (-0.85)	1.27 (0.07)	0.85 (-0.17)	1.15 (0.40)	1.63 (0.49)	0.87 (0.01)	1.74 (0.66)
Unstable living situation	0.00 (-18.42)	0.00 (-19.12)	1.46 (0.38)	1.52 (0.42)	1.00 (0.04)	0.32 (-1.13)	1.21 (0.19)	0.47(-0.75)	8.60 (1.78)**	7.76 (2.01)**
Omnibus test of model coefficients χ2 (p value)	18.23 (0.05)	44.22 (0.00)	23.65 (0.00)	8.64 (0.57)	33.00 (0.00)	8.50 (0.58)	14.38 (0.16)	6.38 (0.78)	25.74 (0.01)	69.73 (0.00)
Nagelkerke R2	0.33	0.35	0.21	0.07	0.13	0.07	0.10	0.15	0.17	0.38

*p ≤ 0.05, **p ≤ 0.01, ^*** ^p ≤ 0.001

^† ^vs. Both long/short-term partners at same time vs. long-term partner only

^‡^p = 0.055

## Discussion

The goal of the parent study (REAL MEN) was to evaluate a multi-dimensional intervention designed to reduce HIV risk and recidivism for young men leaving jail in the context of their actual lives [5,16]. Sex partner experience as defined in this report explored just one of these contextual factors. Prior to incarceration for 16-18-year-old men, having a long-term sex partner is protective against sexually risky behavior, daily marijuana use, and carrying weapons during illegal activity, especially compared to those who have both long-term and short-term sex partners simultaneously. These results build on prior work that demonstrates the potential positive influence of having a romantic relationship for adolescents [6,8]. It is also worth noting that the present study demonstrated that having short-term sex partners only, did not generally increase risk while having both types did.

Over time, the effect of sex partner experience on the outcomes in this study diminished: having a long-term partner no longer looked "less risky," which confirms the argument that "risk" is variable for young people and changes over time and developmental stages [18-20]. While pathways to sex risk behavior are still unclear after the present analysis (although participants seem to be having less sex overall post-incarceration), unemployment and housing instability were important predictors of post-incarceration outcomes, particularly recidivism. Unstable housing was arguably the most important predictor of being rearrested and reincarcerated.

To summarize, while sex partner experience - in particular, having long-term sex partners - may protect young men from some risky sexual behavior, drug use, and violent activity, much larger structural interventions are needed to combat the effects of incarceration, aging, and instability in young people's lives as they move from correctional facilities into the community.

But what is it about having long-term sex partners that seems to protect these young men, at least prior to incarceration? Is it as McCarthy and Casey [8] argue, improved social bonds and increased social control? Perhaps there is a developmental explanation, as well. For young men (the REAL MEN participants were 16-18 at the time of the first interview), having a long-term partner may be protective against negative health and social outcomes. However by age 21, having different types of relationships may become a normative experience and other threats (like being unemployed or living from place-to-place) become more solvent. Perhaps this study encourages further investigation of the role of relationships within young minority men's lives - how do these relationships operate? How are they balanced against other sources of oppression in these young men's lives?

### Limitations of the Present Study

It is important to point out the methodological limitations of the present study. For one, the presence of sex partners was a "yes-no" response to a survey question. Research assistants did not probe participants about the nature of these sex partners. This study does not illustrate how sex partners actually function in the lives of the adolescent men on a day-to-day basis. Second, it is unclear *why *having both long-term and casual sex partners simultaneously represents a risk. Having both types of relationships may be a normative experience for young people, although this may also represent lack of consistent social support and trusting relationships. Third, while the study is able to demonstrate overall differences in type of sex partner at both time points, we have no way of knowing if the sex partner at Time 1 was the same sex partner at Time 2. Fourth, the survey did not assess opposite sex versus same-sex relationships - no such questions were included in the survey because of prior inclusion of such questions resulted in extremely low rates of same-sex relationship reports, suggesting that jail-based interviews may not be a safe place to explore male same-sex behavior. Future research studies may need to include ethnographic investigation in order to find safe and ethical ways to better understand the role of romantic and sexual relationships for incarcerated young men who have sex with male partners.

Another important limitation of this study is the absence of the voices of the young women who are these young men's partners. This study does not consider what the female partners of the young men gain or lose from these relationships. Among adolescents in the free world, Giordano et al. [6] demonstrated that compared to males, females in relationships don't gain as much in school performance. Grinstead et al. [21] showed substantial HIV risk for female partners of incarcerated men. Future research should investigate the experience of these types of long-term relationships for women or other partners of young men involved in the criminal justice system.

## Conclusions

This study illustrates two important findings: sex partner experience matters when it comes to certain health and social risks; and, there seem to be larger structural and policy barriers to success over time for young Black and Latino men involved in the criminal justice system in the US. Programs that serve young men involved in the criminal justice system might incorporate networks of support, particularly sex partnerships, into intervention design. But these programs would also do well to include substantial employment and housing support for easing the transition from jail to community.

## Competing interests

The authors declare that they have no competing interests.

## Authors' contributions

MR was responsible for the conception of this paper, analysis and interpretation of data, and drafting of the manuscript. NF was responsible for the acquisition of funding and data, conception of the study design, reviewing and revising the paper, and giving final approval for the version of this manuscript to be published.

## Pre-publication history

The pre-publication history for this paper can be accessed here:

http://www.biomedcentral.com/1471-2458/10/689/prepub
